# On the Billefjorden fault zone in Garmdalen, central Spitsbergen: implications for the mapping of major fault zones during geological fieldwork and for the tectonic history of Svalbard

**DOI:** 10.12688/openreseurope.17826.1

**Published:** 2024-07-12

**Authors:** Jean-Baptiste P. Koehl, Eirik M. B. Stokmo, Jhon M. Muñoz-Barrera

**Affiliations:** 1 Earth and Planetary Sciences, McGill University, Montreal, Québec, H3A 0E8, Canada; 2 Geosciences, University of Oslo, Oslo, Oslo, 0371, Norway; 3 Rare Earths Norway A.S, Brennebu A.S., Ulefoss, 3830, Norway; 4 National Agency of Hydrocarbon, Bogota, Cundinamarca, 111321, Colombia; 5 Earth Science, University of Bergen, Bergen, Hordaland, 5007, Norway

**Keywords:** Svalbard, Billefjorden Fault Zone, Devonian, fault, thrust, décollement, Svalbardian Orogeny, Eurekan, Carboniferous, Permian, Cenozoic

## Abstract

**Background:**

The present contribution reexamines the geometry of a segment of a presumably long-lived fault in Svalbard, the Balliolbreen Fault segment of the Billefjorden Fault Zone, along which presumably two basement terranes of Svalbard accreted in the early–mid Paleozoic after thousands of kilometers strike-slip displacement.

**Methods:**

We performed structural fieldwork to Billefjorden in central Spitsbergen and interpreted satellite images.

**Results:**

Field observations demonstrate that the Balliolbreen Fault formed as a top-west thrust fault in the early Cenozoic and that weak sedimentary units such as shales of the Lower Devonian Wood Bay Formation and coals of the uppermost Devonian–Mississippian Billefjorden Group partitioned deformation, resulting in significant contrast in deformation intensity between stratigraphic units. For example, tight early Cenozoic folds are localized in shales of the Wood Bay Formation and contemporaneous top-west brittle–ductile thrusts within coals of the Billefjorden Group, whereas Pennsylvanian deposits of the Hultberget (and/or Ebbadalen?) Formation are simply folded into gentle open folds. Rheological contrasts also resulted in the development of décollements locally, e.g., between tightly folded strata of the Wood Bay Formation and Billefjorden Group and flat-lying, brecciated limestone-dominated strata of the Wordiekammen Formation. Despite the limited quality and continuity of outcrops in the area, the eastward-thickening character (i.e., away from the fault) of Pennsylvanian deposits of the Hultberget, Ebbadalen, and Minkinfjellet formations suggests that the fault did not act as a normal fault in Pennsylvanian times.

**Conclusions:**

The study suggests that strain partitioning of early Cenozoic Eurekan contraction alone may explain the deformation patterns in Paleozoic rock units in central Spitsbergen, i.e., that Late Devonian Svalbardian contraction is not required, and that a major segment of the Billefjorden Fault Zone formed in the early Cenozoic. The present work illustrates the crucial need for interdisciplinary approaches and composite educational backgrounds in science.

## Plain Language Summary

The present study reexamines the geometry, kinematics, and tectonic history of a major fault zone in the Norwegian Arctic, the Balliolbreen Fault segment of the Billefjorden Fault Zone. Thus far, the latter fault was believed to represent a hundreds to thousands of kilometers long discontinuity in the crust, possibly linking with major faults in northern Scotland (e.g., Great Glen Fault). In addition, it was believed to have accommodated hundreds to thousands of kilometers of lateral displacement between 500 Ma and 400 Ma based on similar rock types in northern Scotland and northern Spitsbergen and between northeastern Svalbard and northern Greenland, thus suggesting discrete origins for the crustal blocks constituting the Svalbard Archipelago.

Our results and recent studies show that the Balliolbreen Fault formed c. 65–30 million years ago, i.e., much later than previously proposed, that it dips gently to the east (instead of steeply), and that it accommodated a limited amount of vertical (instead of large lateral) tectonic movements. The fault likely formed between weak Lower Devonian (c. 420-390 million years old) rock units, which consist of fine-grained rocks easily deformed into folds during tectonic events, and strong Pennsylvanian-Permian (c. 330-290 million years old) rock units, which consist of coarse-grained rocks that do not deform easily and, instead, rupture once enough tectonic movement has occurred. The study has implications for how geoscientists map major faults during fieldwork and how they interpret structural datasets and extensively eroded outcrops transects. It also suggests that the Svalbardian Orogeny, a major episode of tectonic contraction, which presumably occurred c. 380-370 million years ago in Arctic regions, did not occur. The study calls for more interdisciplinary approaches when performing scientific research, especially when mapping major fault zones during geological fieldwork. It also highlights the need of more composite educational backgrounds for researchers.

## Introduction and regional geology

**Figure 1.**
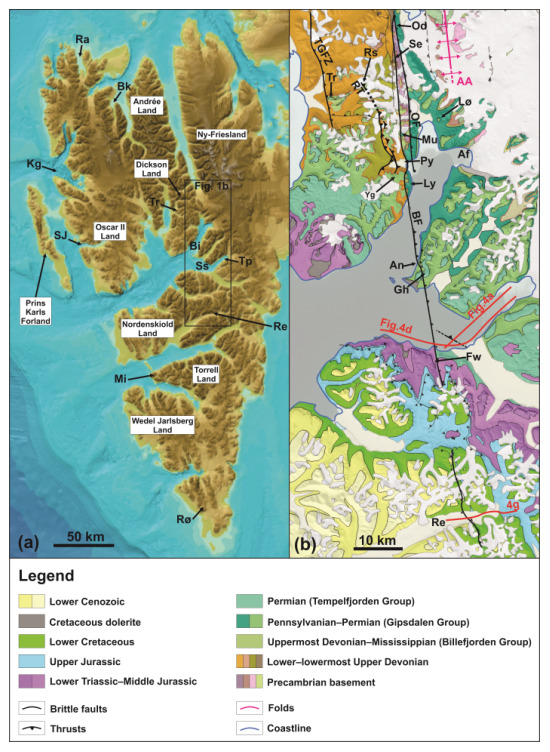
( **a**) Overview of the study area in central Spitsbergen showing the extent of the Billefjorden Fault Zone based on previous studies. Modified after Jakobsson *et al.* (2012). ( **b**) Geological map of central Spitsbergen modified from geokart.npolar.no and Koehl (2021) showing the various segments of the Billefjorden Fault Zone and the location of the study area (Garmdalen; the yellow rectangle shows the location of Figure 2). Abbreviations: AA: Atomfjella Antiform; Au: Austfjorden; Av: Alvrekdalen; BaF: Balliolbreen Fault; Bb: Brucebyen; BFZ: Billefjorden Fault Zone; Cp: Cowantoppen; Gd: Garmdalen; GF: Gipshuken Fault; Gh: Gipshuken; Gk: Garmaksla; Mb: Mimerbukta; Md: Munindalen; Mu: Mumien; Oa: Odindalen; OF: Odellefjellet Fault; Py: Pyramiden; Se: Sentinelfjellet; Wi: Wijdefjorden; Wo: Wordiekammen; Yg: Yggdrasilkampen.

The Billefjorden Fault Zone ( Figure 1) is commonly interpreted as an asymmetric half-graben boundary fault ( Braathen *et al*., 2011; Dallmann, 1993; Johannessen & Steel, 1992; McCann & Dallmann, 1996; Pickard *et al*., 1996; Smyrak-Sikora *et al*., 2018). In addition, stepping and overlapping attitudes between its various segments suggest strike-slip behavior to some extent. For example, the Odellfjellet Fault dies out southwards in Mimerbukta steps into the Balliolbreen Fault ( Braathen *et al*., 2011; Dallmann *et al*., 2004; Figure 1). Strike-slip behavior was also inferred for the accretion of Svalbard’s three basement terranes in the Paleozoic through hundreds–thousands of kilometer-scale lateral movements along N–S-striking faults like the long-lived Billefjorden Fault Zone ( Harland, 1969; Harland *et al*., 1974; Harland *et al*., 1992; Harland & Wright, 1979; Labrousse *et al*., 2008).

Although an origin as an early–mid Paleozoic strike-slip fault or as a Late Devonian Svalbardian thrust are rather unlikely (e.g., Koehl, 2021; Koehl, 2024; Koehl & Allaart, 2021; Koehl *et al*., 2022a; Koehl *et al*., 2022b; Koehl *et al*., 2023a; Koehl *et al*., 2023b; Lamar *et al*., 1986), normal, rift-related movements along the western boundary of the Billefjorden Trough are still commonly proposed to explain the observed fault geometries and stratigraphic relationships in the field (e.g., Braathen *et al*., 2011; Cutbill *et al*., 1976; Johannessen & Steel, 1992; Smyrak-Sikora *et al*., 2018). Nevertheless, despite numerous studies of the Billefjorden Fault Zone (e.g., Braathen *et al*., 2011; Bælum & Braathen, 2012; Harland *et al*., 1974; Koehl, 2021; Koehl & Allaart, 2021; Lamar *et al*., 1982; Lamar *et al*., 1986; Lamar & Douglass, 1995; Smyrak-Sikora *et al*., 2018), several aspects of its tectonic history remain unclear. Notably, the Balliolbreen Fault displays significant along-strike variations in both inferred kinematics, apparent cross-section geometry, amount of offset, and time of formation. For instance, the Balliolbreen Fault was interpreted as a moderately to steeply dipping, Late Devonian, top-west reverse fault in Odellfjellet, Sentinelfjellet, and Garmdalen ( Dallmann & Piepjohn, 2020; Harland *et al*., 1974; Lamar *et al*., 1986; Lamar & Douglass, 1995; see locations in Figure 1), whereas the fault reflects low-angle, top-west Eurekan thrusting, including (bedding-parallel) duplexes and link thrusts ( Koehl, 2021), and, potentially, high-angle Carboniferous normal faulting in Pyramiden ( Braathen *et al*., 2011; Smyrak-Sikora *et al*., 2018; location shown in Figure 1).

A major issue in inferring the kinematic, geometry and formation timing of the Balliolbreen Fault is related to the poor quality and limited extent of outcrops in Svalbard. Key outcrops that could help unravelling these relationships are discontinuous because largely eroded and mostly covered by loose material. Other key outcrops are located on steep slopes, which are difficult to access and therefore hamper/prevent detailed inspection of the fault and of stratigraphic contacts (see toposvalbard.npolar.no for aerial photographs of outcrop transects in central Spitsbergen). Notably, high uncertainties remain regarding, e.g., the truncation or not of the Billefjorden Group by the fault and, thus, on the actual timing of faulting in Sentinelfjellet ( Lamar *et al*., 1982; Lamar *et al*., 1986; Lamar & Douglass, 1995), and the occurrence of normal faulting along the Balliolbreen Fault in Pyramiden ( Koehl, 2021).

The main segments of the Billefjorden Fault Zone include, from north to south, the Odellfjellet Fault (Odellfjellet to Pyramiden), the Balliolbreen Fault (Odellfjellet to Garmaksla), and the Gipshuken Fault (Cowantoppen to Gipshuken; Harland *et al*., 1974; Figure 1). Farther south in Nordenskiöld Land, the Billefjorden Fault Zone dips gently to the east and is thought to truncate and offset Paleozoic–Mesozoic sedimentary successions top-west ( Haremo, 1989; Haremo, 1992; Haremo *et al*., 1990; Major & Nagy, 1972; Figure 1), and recent work on seismic data suggest that the fault accommodated exclusively early Cenozoic contraction ( Koehl, 2021).

**Figure 2.**
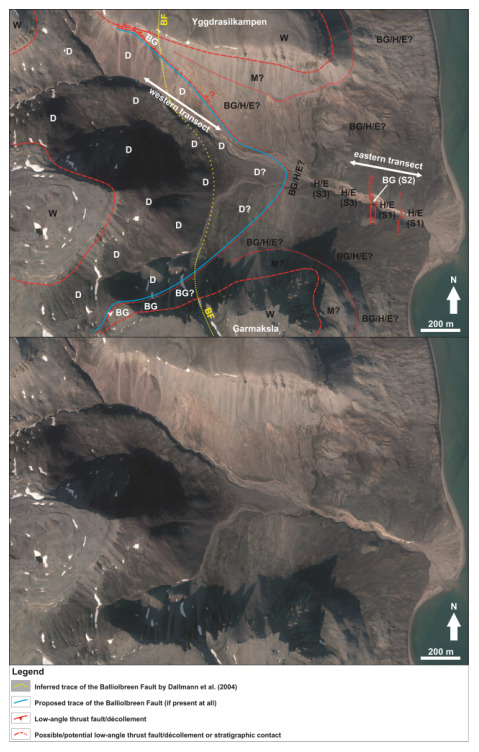
Interpreted (up) and uninterpreted (down) aerial photograph from toposvalbard.npolar.no showing the observed and inferred lithostratigraphic contacts and faults in Garmdalen. Letters with question mark denote loose material (e.g., screes), whereas letters without question mark denote in-place bedrock. Abbreviations: BF: Balliolbreen Fault; BG: Billefjorden Group; D: Lower Devonian Wood Bay Formation; E: Ebbadalen Formation; H: Hultberget Formation; M: Minkinfjellet Formation; S1: Succession 1; S2: Succession 2; S3: Succession 3; W: Wordiekammen Formation.

**Figure 3.**
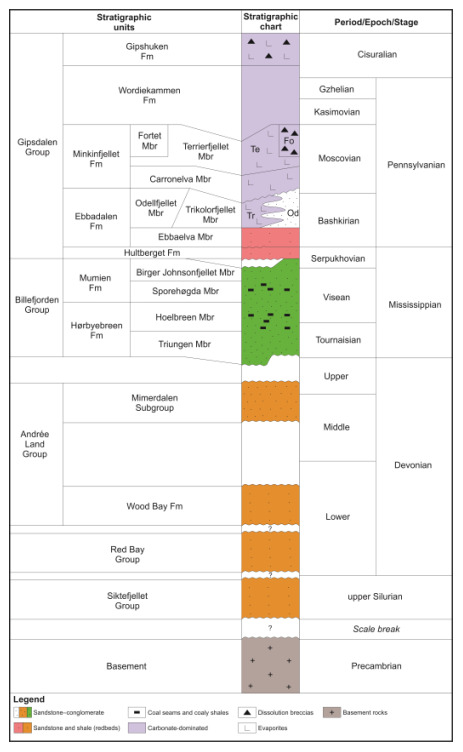
Lithostratigraphic chart of mid–late Paleozoic sedimentary rocks in Spitsbergen. The chart is based on work by Aakvik (1981); Braathen *et al*. (2011); Cutbill & Challinor (1965); Cutbill *et al*. (1976); Dallmann & Piepjohn (2020); Dallmann *et al*. (1999); Friend *et al*. (1966); Friend *et al*. (1997); Friend & Moody-Stuart (1972); Gee *et al*. (1952); Gee & Moody-Stuart (1966); Gjelberg (1983); Gjelberg (1984); Gjelberg & Steel (1981); Holliday & Cutbill (1972); Johannessen (1980); Johannessen & Steel (1992); Lønøy (1981); Lønøy (1995); McWhae (1953); Murascov & Mokin (1979), and Playford (1962).

**Figure 4.**
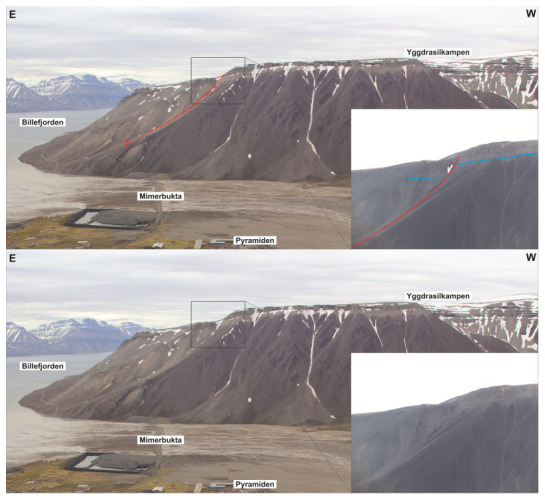
Interpreted (up) and uninterpreted (down) view of the Balliolbreen Fault segment of the Billefjorden Fault Zone in southern Mimerdalen–Yggdrasilkampen (red lines). The fault downthrows the base of the uppermost Pennsylvanian–lower Permian Wordiekammen Formation to the east (blue lines). We reinterpret the offset of the base Wordiekammen Formation as a recent (Quaternary?) landslide due to strong inconsistencies with the Balliolbreen Fault in Garmdalen.

South of Mimerbukta, the Balliolbreen Fault is thought to proceed onto Yggdrasilkampen, Garmdalen, and Garmaksla ( Figure 1 and Figure 2) where it was interpreted as a Late Devonian high-angle reverse fault and/or (reactivated as) a Carboniferous–Permian normal fault ( Dallmann *et al*., 2004; Dallmann *et al*., 2009; Dallmann & Piepjohn, 2020). The Late Devonian reverse fault interpretation was inferred from intense contractional deformation of Lower Devonian rocks of the Wood Bay Formation ( Figure 3) in the footwall of the fault. The interpretation as a Carboniferous–Permian normal fault is based on the presence of hundreds of meters thick Pennsylvanian strata of the Hultberget, Ebbadalen and Minkinfjellet formations ( Figure 3) in the hanging wall but not in the footwall, and on an apparent 10–15 meters large down-east normal offset of the base of the Wordiekammen Formation ( Figure 3) on the Yggdrasilkampen mountain top in southern Mimerdalen ( Dallmann *et al*., 2004; Figure 4). We re-examine the evidence and further discuss structural and stratigraphic relationships in this area.

The results of the present field study have implications on the mapping of major fault zones during geological fieldwork, which are too often inferred from insufficient data and used as quick fixes for gaps in our understanding of the geology of an area. Other implications include strain partitioning processes, e.g., during early Cenozoic Eurekan contraction in Svalbard (e.g., Dallmann *et al*., 1993), and the Late Devonian Svalbardian Orogeny in Spitsbergen ( Harland *et al*., 1974; McCann, 2000; Piepjohn, 2000; Vogt, 1928; Vogt, 1938). Potential implications for structural inheritance in central Spitsbergen are discussed, including the influence of Carboniferous normal faults and Caledonian folds and thrusts ( Witt-Nilsson *et al*., 1998). The present study has also a broader significance for Phanerozoic plate reconstructions, especially for the tectonic model commonly used to explain terrane accretion in Svalbard in the early–mid Paleozoic ( Harland, 1969; Harland *et al*., 1992; Labrousse *et al*., 2008), suggesting that Svalbard’s terranes (if they indeed are terranes) were accreted earlier than the Paleozoic.

## Methods

We performed structural fieldwork in Billefjorden, Svalbard, in summer 2021 ( Figure 1) and interpreted satellite images from the Norwegian Polar Institute in the area. During fieldwork, we overnighted at the Pyramiden Hotel and had daily trip to the outcrops from Pyramiden using a rubber boat rented from the Norwegian Polar Institute in Longyearbyen.

Since the study of cleavage is not a discriminating factor to segregate tectonism events from one another (no matter the number of structural measurements; e.g., Fossen *et al*., 2019; Ghosh *et al*., 1995; Johnson & Woodcock, 1991; Morley, 1986; Oriolo *et al*., 2022; Treagus & Treagus, 1992), we did not include any such measurements in the present study. The structural data and field photographs are available on DataverseNO ( doi.org/10.18710/TIIIKX and doi.org/10.18710/BIJYVO).

The figures were designed using CorelDraw 2017 (open-source alternative: GIMP) and Hugin Panorama photo stitcher 2018.0 to create panorama of large outcrops.

## Results

### Eastern transect in garmdalen

***Description.*** In eastern Garmdalen (see Figure 1 for location), we investigated three sedimentary successions that have not yet been studied in detail. The easternmost succession (succession 1) is comprised of gently east dipping and mildly folded strata dominated by yellow sandstone and red and grey shale and siltstone ( Figure 4a–c). The shales display evidence of bedding-parallel brittle–plastic shearing appearing in the form of S- and Z-shaped brittle–ductile fractures arranged in duplex- to imbricate-like geometries and dying out at the top and base of shale-rich beds ( Figure 4d–e). Both sandstone, shale and siltstone beds are deformed into N–S-striking, open fold structures ( Figure 4a–b).

In the west, succession 1 overlies succession 2, which corresponds to a several meters thick unit of strongly plastically deformed, dark, coal-rich, phyllitic shales ( Figure 6a) with thin, S-shaped layers of light-colored quartzite ( Figure 6b; see Figure 2 for location of succession 2). This succession includes centimeter–meter-scale angular to sub-rounded, in places sigmoid-shaped clasts of dark to yellow sandstone and quartzite (in fuchsia in Figure 6c).

Farther west, the strongly deformed, dark, coal-rich shales overlie a succession consisting of gently to moderately east-dipping yellow sandstone, red and grey shales and siltstone (succession 3; Figure 6d–e; see Figure 2 for location of succession 3). The red and grey shales appear strongly deformed, displaying numerous small-scale brittle–ductile fractures/shears, whereas the yellow sandstones and siltstones display only a few brittle fractures with no offset ( Figure 6f).

Up the northern and southern mountain flanks, outcrops are replaced by red to grey screes and loose material, which are overlain by several meters thick carbonate beds on the mountain tops in Yggdrasilkampen and Garmaksla ( Figure 2; see Figure 1 for location). The carbonate beds are possible to correlate regionally to the Wordiekammen Formation ( Dallmann *et al*., 2004).

***Interpretation.*** The enrichment in coal of succession 2 ( Figure 6a–e) and the occurrence of characteristic cliff outcrops of carbonate beds of the Wordiekammen Formation on the mountain tops in Garmdalen both to the north and south ( Figure 2), i.e., well above succession 2, indicate that succession 2 is part of the Billefjorden Group. This is supported by the presence of dark–yellow sandstone, coal, and coaly shale of the Billefjorden Group at the same altitude (i.e., near sea-level) in adjacent areas (e.g., Brucebyen) and near the Balliolbreen Fault (e.g., Pyramiden; see Figure 1 for location; Aakvik, 1981; Cutbill *et al*., 1976; Koehl, 2021).

The intense plastic deformation of the dark, coal-rich shales in the Billefjorden Group (succession 2) suggests that thee observed sigmoidal clasts of sandstone (e.g., pink lines in Figure 6a and c) correspond to sigma-clasts and that S-shaped layers/lenses of light-colored quartzite (white lines in Figure 6a–e) represent asymmetric, centimeter–decimeter-scale pinch-and-swell structures and duplexes (e.g., Boyer & Elliott, 1982) reflecting top-west tectonic transport. Notice that tectonic transport within the Billefjorden Group occurs near-parallel to the gently east-dipping bedding surfaces ( Figure 6a–e).

Because of the strong similarities, both in the dominant lithologies and deformation character, and based on top-west contractional structures within the Billefjorden Group, we correlate the yellow sandstones and grey and red shales and siltstones overlying and underlying the Billefjorden Group (succession 2) as part of the same succession (successions 1 and 3; Figure 7). Top-west contractional structures within the Billefjorden Group indicate that succession 3 corresponds to the offset counterpart of upthrust succession 1 (i.e., tectonic repetition; Figure 7). Since succession 1 lies directly over the Billefjorden Group in the east, we interpret successions 1 and 3 as parts of the Hultberget Formation. This is in agreement with previous interpretations by Smyrak-Sikora *et al*. (2018) and Senger *et al*. (2018) in Garmdalen.

Our correlation of Hultberget Formation both over (i.e., normal stratigraphy) and under the Billefjorden Group (reversed stratigraphy) suggests the presence of a gently east-dipping thrust fault between succession 2 and 3, i.e., at the base or within the Billefjorden Group ( Figure 7). The amount of tectonic offset along the thrust is most likely in the order of at least a few tens of meters to a hundred meters. This is suggested by tectonic repetition of the Hultberget Formation, and by the very thin character of the Billefjorden Group in eastern Garmdalen (a few meters thick; Figure 6a–f and Figure 7), which is much thinner than the typical thickness of Billefjorden Group deposits in adjacent areas in Billeforden such as Pyramiden (c. 300 meters) and Brucebyen (c. 100 meters; Aakvik, 1981; Cutbill *et al*., 1976).

**Figure 5.**
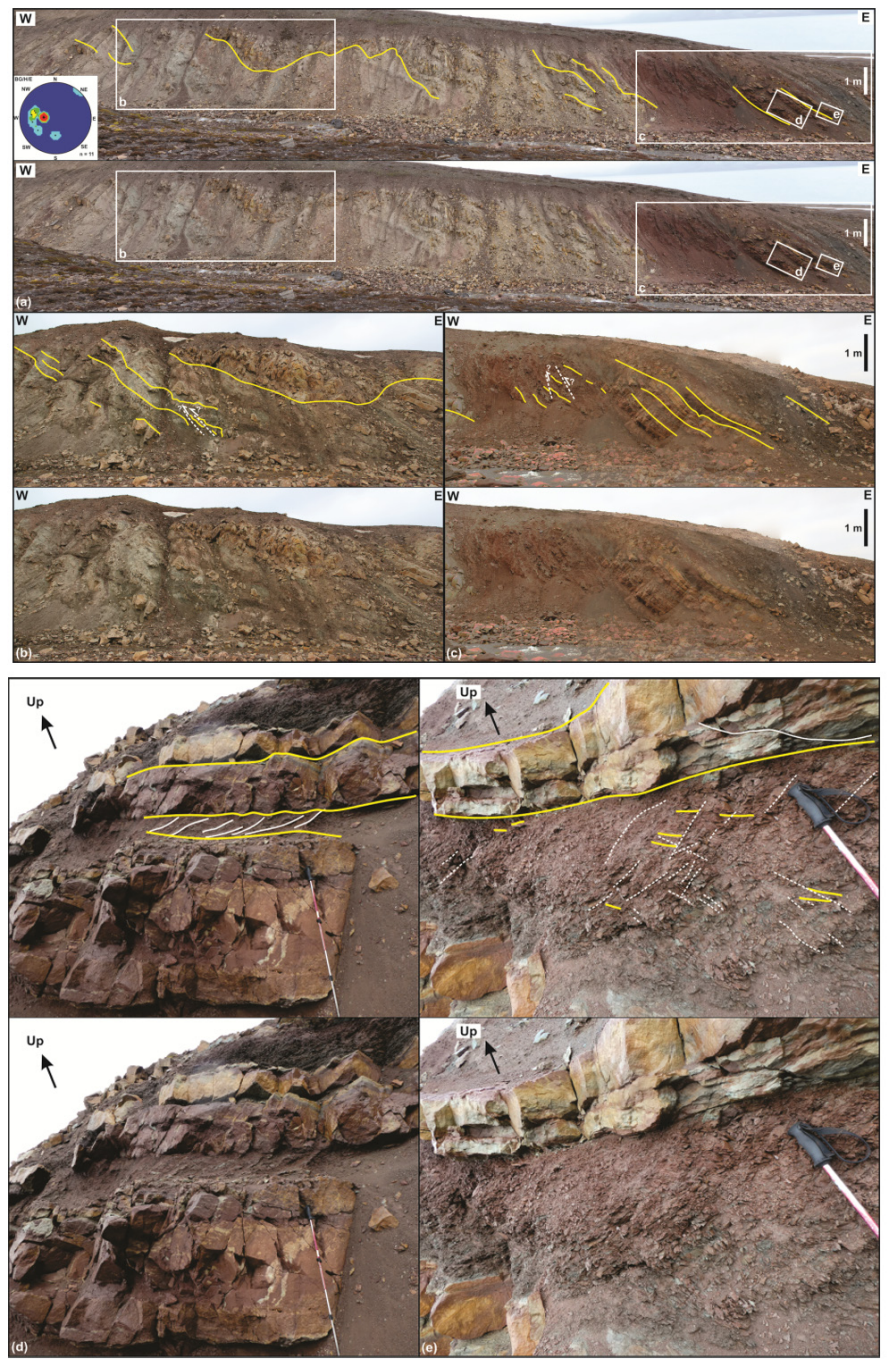
Interpreted (up in each inset) and uninterpreted (down in each inset) outcrop photographs of succession 1 in eastern Garmdalen showing gently east-dipping, interbedded grey and red shales–siltstone and yellow sandstone strata most likely of the Hultberget Formation. The strata are deformed into gentle, open N–S-trending folds ( **a**), and possibly crosscut by a few, minor reverse faults with centimeter–meter-scale top-west offset (dashed white lines in **b** and **c**). The stereonet in ( **a**) shows bedding surfaces within the Billefjorden Group and Hultberget (and/or Ebbadalen?) Formation. ( **d**) and ( **e**) illustrate differential deformation between poorly deformed blocky beds of yellow sandstone, which are crosscut by a few fractures displaying no apparent offset, and grey and red shales, which are truncated by numerous brittle fractures (see white lines in ( **d**)) and possible S-C ductile fabrics (see dashed white lines and yellow lines within the red–grey shale interval in ( **e**)). Photos ( **d**) and ( **e**) by Erik Johannessen.

**Figure 6.**
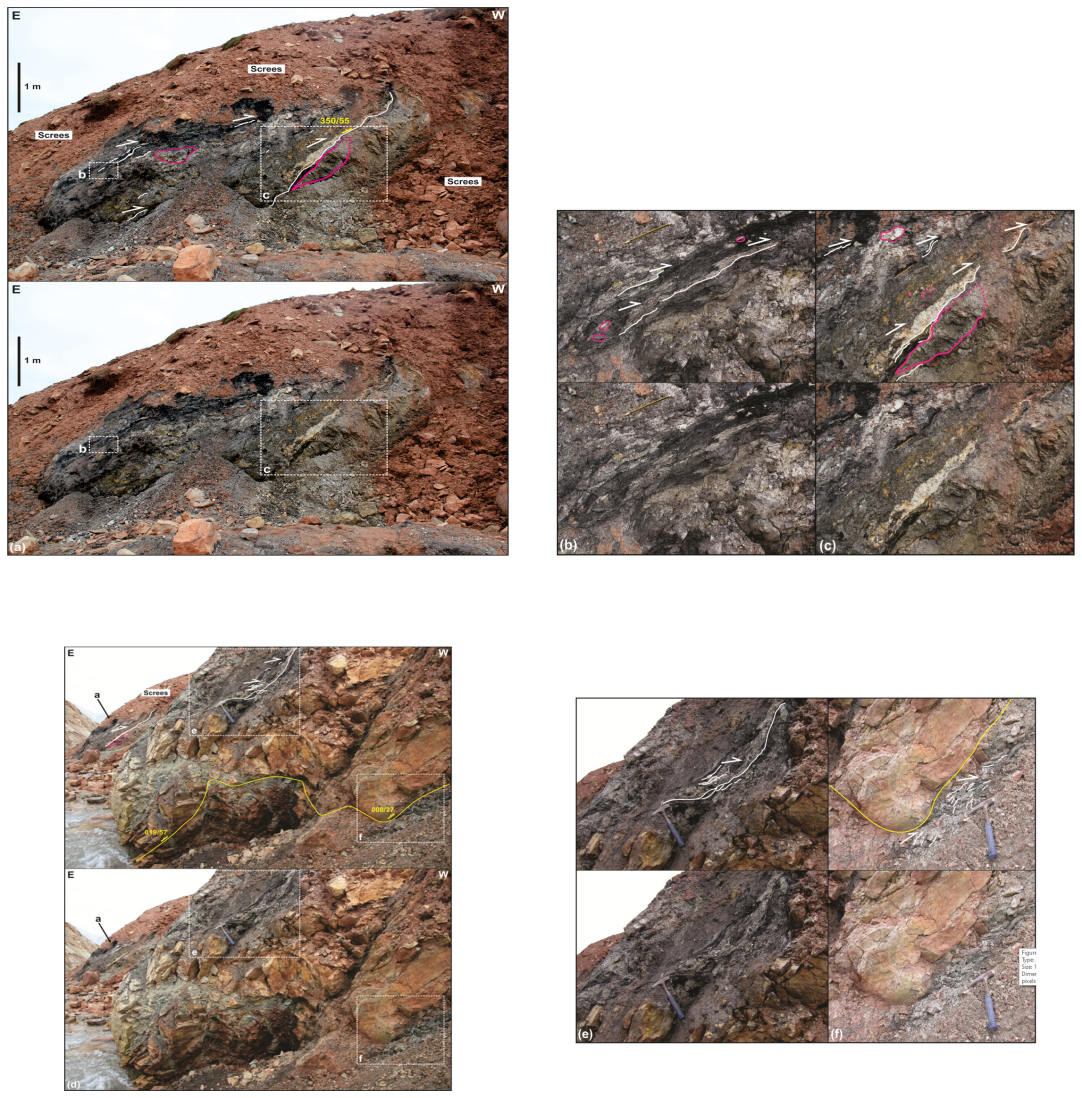
Interpreted (up in each inset) and uninterpreted (down in each inset) outcrop photographs of successions 2 and 3 in eastern Garmdalen. ( **a**) Tectonized coaly shales, coals, quartzite and dark sandstone of succession 2, most likely belonging to the Billefjorden Group. Succession 2 is comprised of ( **b**) intensely sheared quartzite and coal intervals displaying S-shaped fabrics such as pinch-and-swell structures (white lines) indicating top-west thrusting, and ( **c**) of angular to sub-rounded, centimeter–meter-scale blocks of dark sandstone in place showing sigma-clast-like shapes (e.g., lower block highlighted by pink lines). ( **d**) Tectonized contact between successions 2 (Billefjorden Group) and 3 (Hultberget Formation). Notice the location of the outcrop shown in ( **a**) in the upper left corner in ( **d**) (the meter-scale block of dark sandstone is the same as in ( **a**)). The outcrop in ( **d**) shows near bedding-parallel, S-shaped brittle–ductile fabrics indicating top-west thrusting within coals and quartzite layers of succession 2 (see also white lines in ( **e**)), and mildly folded, gently–moderately east-dipping strata of yellow sandstone and intensely deformed red–grey shale and siltstone (with brittle–ductile shear fabrics; see white lines in (f)) of succession 3.

Since both the Billefjorden Group and Hultberget Formation are deformed, the thrust fault within the Billefjorden Group ( Figure 6a–f) and associated N–S-trending folds in the Hultberget Formation ( Figure 5a–c) formed in the early Cenozoic, i.e., during Eurekan contraction.

**Figure 7.**
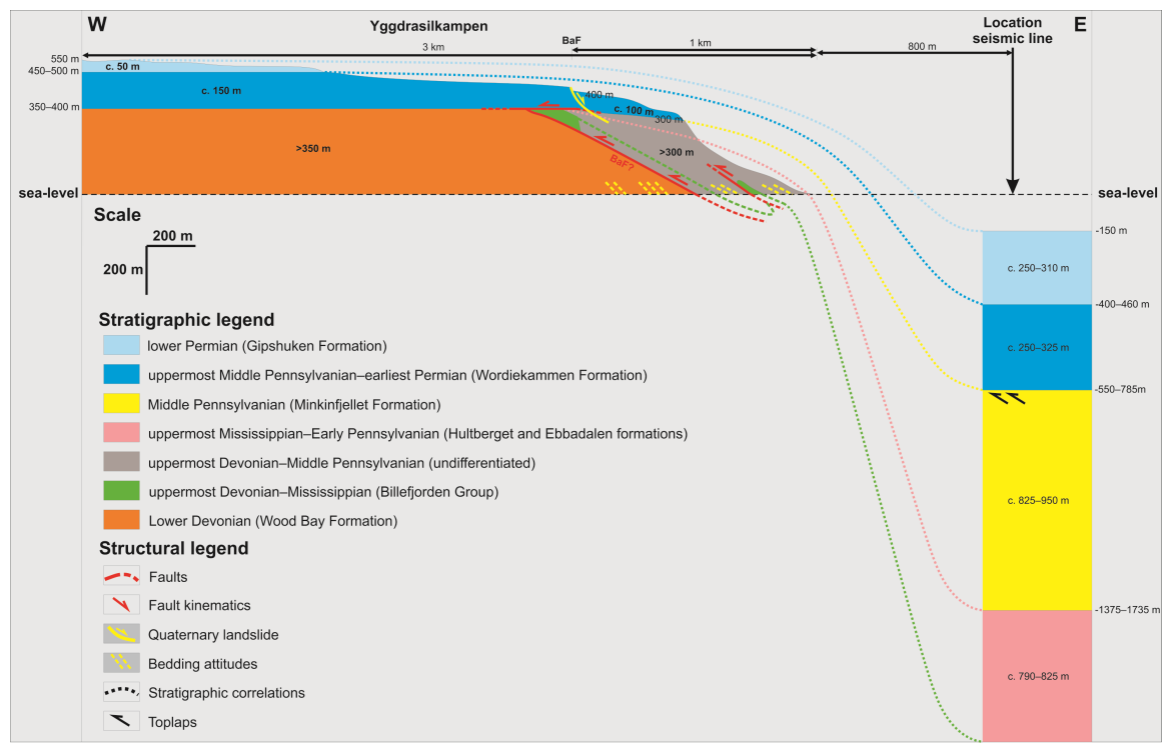
Correlation of the onshore outcrops at Garmdalen and Lykteneset (present study) and offshore interpretation of seismic reflection data in Billefjorden ( Koehl *et al.*, 2023b). The vertical and horizontal scales are the same.

Because they are located above the outcrops of Billefjorden Group and Hultberget Formation and below those of Wordiekammen Formation, grey to red screes in eastern Garmdalen are believed to represent strata of the (upper part of the) Hultberget Formation, and of the Ebbadalen and Minkinfjellet formations covered by loose material ( Figure 2 and grey-shaded area in Figure 7).

### Western transect in garmdalen

**Figure 8.**
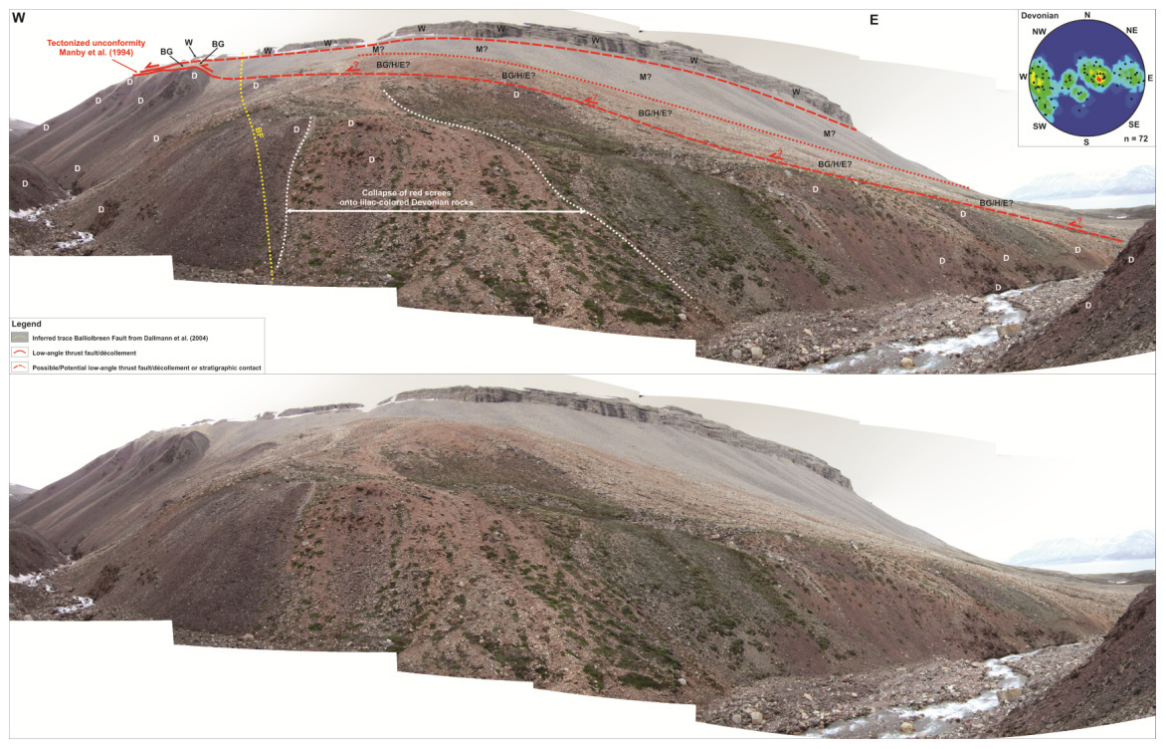
Interpreted (up) and uninterpreted (down) west–east outcrop transect of the northern flank of Garmdalen/southern Yggdrasilkampen. The transect shows the cross-section geometry of the Balliolbreen Fault (BF) in northern Garmdalen inferred by previous works and by the present study. Letters with question mark denote loose material (e.g., screes), whereas letters without question mark denote in-place bedrock. Notice the sharp contrast between the high-angle Carboniferous normal fault geometry of Dallmann *et al.* ( 2004; dotted yellow line) and the low-angle Cenozoic thrust geometry (if present at all) proposed by the present study (red line). Note that a high-angle Carboniferous normal fault in the area is highly unlikely due to the absence of apparent offset of Devonian sedimentary rocks (D) across the inferred trace of the Balliolbreen Fault (dotted yellow line). The normal fault geometry is possibly a misinterpretation due to the collapse of light red screes of the Hultberget and/or Ebbadalen formations over outcrops of lilac-colored Devonian rocks. The stereonet in the upper right corner shows bedding surfaces within the Lower Devonian Wood Bay Formation. Notice how Devonian sedimentary rock appear folded into overall N–S-trending, sub-horizontal folds. Abbreviations: BF: Balliolbreen Fault; BG: Billefjorden Group; D: Lower Devonian Wood Bay Formation; E: Ebbadalen Formation; H: Hultberget Formation; M: Minkinfjellet Formation; W: Wordiekammen Formation.

***Description.*** In western Garmdalen, outcrops are of much lower quality and less continuous but show lilac- and green-colored, gently to steeply east- and west-dipping ( Figure 8) shales and siltstone–sandstone, some of which are partly covered by red-colored screes and loose material. The strata by the riverbed are typically deformed into tight, N–S-trending folds. No trace of brittle deformation such as cataclasis or brecciation was identified in lilac- and green-colored rock units. A few isolated outcrop patches of lilac- and green-colored, folded siltstone–sandstone and shale crop out up the slope over the riverbank in northwestern Garmdalen. These isolated outcrops are surrounded by red screes, which cover most of the mountain flanks ( Figure 8). Outcrops of lilac-colored shale and siltstone–sandstone are found gradually farther up the mountain westwards and occur in contact with yellow sandstones of the Billefjorden Group and flat-lying rocks of the Wordiekammen Formation in inner Garmdalen ( Figure 2 and Figure 7; Manby *et al*., 1994 their figure 12). The unconformities between these three units (lilac-colored shale and siltstone–sandstone, Billefjorden Group, and Wordiekammen Formation) are tectonized and both the sandstones of the Billefjorden Group and carbonates of the Wordiekammen Formation are brecciated ( Figure 7 and Manby *et al*., 1994 their Figure 12).

In between innermost and eastern Garmdalen, lilac- and green-colored shales and siltstones–sandstones are replaced by red screes along the riverbed ( Figure 2, Figure 8, and grey-shaded area in Figure 7). Half-way up the mountain flank, red screes are replaced by grey–brown screes, which are overlain by blocky and continuous outcrops of carbonates of the Wordiekammen Formation ( Figure 2, Figure 8, and grey-shaded area in Figure 7). Notice how red and grey–brown screes pinch out upwards and westwards ( Figure 8 and grey-shaded area in Figure 7) towards the area where lilac-colored shales are in contact with tectonized sandstone of the Billefjorden Group and brecciated carbonates of the Wordiekammen Formation ( Manby *et al*., 1994 their Figure 12).

***Interpretation.*** Because of the low extent and poor quality of outcrops, the geology of the western part of Garmdalen is more difficult to interpret. However, given the stratigraphically low position of the succession of gently to steeply dipping, lilac- and green-colored shale and siltstone–sandstone compared to the successions in eastern Garmdalen, and the notable color difference with red-colored rocks of the Hultberget Formation in the east, we are inclined to agree with previous studies (e.g., Dallmann *et al*., 2004; Dallmann & Piepjohn, 2020) interpreting lilac–green sedimentary rocks as part of the Lower Devonian Dicksonfjorden Member of the Wood Bay Formation (orange-shaded area in Figure 7). This is also supported by the presence of sandstones of the Billefjorden Group over these rocks in inner Garmdalen in the west ( Figure 2, green-shaded area in Figure 7, and Manby *et al*., 1994 their Figure 12).

Because of the absence of outcrops between rocks of the Wood Bay Formation and the Wordiekammen Formation (except in innermost Garmdalen), the bedrock covered by red and grey–brown screes is uncertain. Nonetheless, considering the similar red color of the lower band of screes with that of siltstones of the Hultberget Formation in eastern Garmdalen ( Figure 5a–c and Figure 8) and with strata of the Ebbadalen Formation elsewhere in Billefjorden ( Braathen *et al*., 2011; Johannessen, 1980; Johannessen & Steel, 1992), the red screes in western Garmdalen are believed to reflect the presence of sedimentary rocks of the Hultberget and Ebbadalen formations (grey-shaded area in Figure 7). This is also supported by the alignment of outcrops of gently east-dipping strata of the Hultberget Formation in eastern Garmdalen with the band of red-colored screes in western Garmdalen, which may correspond to the westward continuation of gently east-dipping strata of red beds of the Hultburget and/or Ebbadalen formations in western Garmdalen ( Figure 2). Analogously, overlying grey–brown screes are interpreted to reflect the occurrence of strata of the Minkinfjellet Formation (grey-shaded area in Figure 7), which is present just below carbonates of the Wordiekammen Formation along the shore between Garmdalen and Mimerbukta ( Senger *et al*., 2018).

## Discussion

### The Balliolbreen Fault in Garmdalen

There is no outcrop of Hultberget, Ebbadalen and Minkinfjellet formations in western Garmdalen ( Figure 2 and Figure 8). The nature of the westwards pinch-out of these stratigraphic units is therefore tentative. The contact between Lower Devonian–Mississippian rocks (Wood Bay Formation and Billefjorden Group) and uppermost Mississippian–Pennsylvanian rocks (Hultberget, Ebbadalen, Minkinfjellet, and Wordiekammen formations) in western Garmdalen could either be (1) an angular unconformity with strata of the Billefjorden Group and of the Hultberget, Ebbadalen, and Minkinfjellet formations onlapping onto Lower Devonian–Mississippian strata of the Wood Bay Formation and Billefjorden Group in the west (i.e., eroded prior to the deposition of the Wordiekammen Formation or never deposited), or (2) a top-west, low-angle, bedding-parallel Eurekan thrust–décollement ( Figure 7). A sedimentary origin is suggested by the westward pinching-out attitude of beds of the Billefjorden Group ( Koehl, 2021 his Figure 3) and of the Hultberget, Ebbadalen, and Minkinfjellet formations in the nearby area of Pyramiden ( Smyrak-Sikora *et al*., 2018 their Figure 7). However, it is also at least partly related to tectonic thinning (smearing) due to Eurekan folding and thrusting. This is based on evidence of top-west Eurekan thrusting in coal-rich shales of the Billefjorden Group in eastern Garmdalen ( Figure 6a–e and Figure 7), on relatively intense deformation of shale-rich beds within the Hultberget (and/or Ebbadalen?) Formation compared to sandstone units ( Figure 5d–e and Figure 6f), on the tectonized (brecciated) stratigraphic contacts between the Wood Bay Formation, Billefjorden Group, and Wordiekammen Formation in innermost Garmdalen ( Figure 2 and Manby *et al*., 1994 their Figure 12), and on top-west Eurekan thrusting of the Minkinfjellet Formation onto the Hultberget Formation along the east-dipping Mimerbukta fault along the shore between Garmdalen and Mimerbukta ( Senger *et al*., 2018).

**Figure 9.**
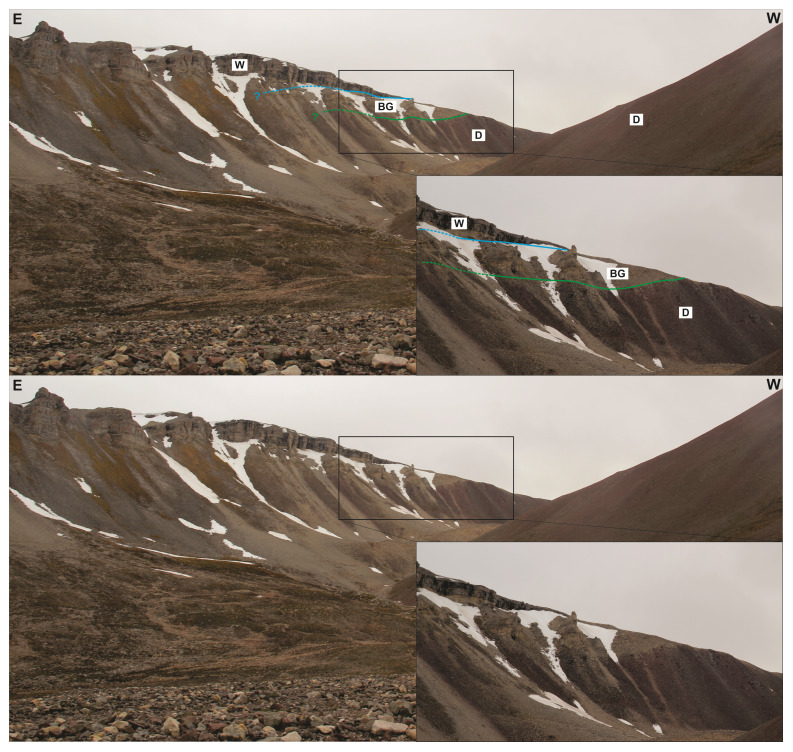
Interpreted (up) and uninterpreted (down) field photograph of the southern flank of Garmdalen showing yellow sandstone possibly representing erosional remnants of the Billefjorden Group (BG; Dallmann *et al*., 2004) overlying the Wood Bay Formation (D) and overlain by the Wordiekammen Formation (W).

Furthermore, sheared, coal-rich shales of the Billefjorden Group are absent both in northwestern and southwestern Garmdalen in between tightly folded rocks of the Wood Bay Formation and brecciated strata of the Wordiekammen Formation ( Figure 9; Manby *et al*., 1994 their Figure 12) and above the coal mine entrance in Pyramiden ( Koehl, 2021). On the contrary, they are present and intensely deformed in eastern Garmdalen ( Figure 6a–e) and below the mine entrance in Pyramiden ( Koehl, 2021). These observations suggest that they were potentially tectonically squeezed out to the east (smeared) during early Cenozoic top-west thrusting. We propose a similar tectonic mechanism (though to a lesser extent) to partly explain the pinching out character of the Hultberget, Ebbadalen and Minkinfjellet formations in western Garmdalen.

There is no evidence of brittle-fault-related deformation or offset, nor of any clear major fault surface at the location of the Balliolbreen Fault in Garmdalen inferred in previous studies as the area is entirely covered by screes (e.g., Dallmann *et al*., 2004; Dallmann & Piepjohn, 2020; see dotted yellow line in Figure 2 and Figure 8). Thus, we propose that the Balliobreen Fault in Garmdalen (if present at all) corresponds to a low-angle, bedding-parallel brittle thrust fault running near or at the contact between the Wood Bay Formation and overlying (mostly scree-covered) rocks of the Billefjorden and Gipsdalen groups in western Garmdalen (see blue line in Figure 2 and lower dashed red line in Figure 8) that is similar to the related low-angle brittle thrust faults (e.g., in eastern Garmdalen; Figure 2, Figure 6a–e, and red lines in Figure 7). The fault is therefore believed to have initiated as an early Cenozoic, top-west Eurekan thrust, which partially decoupled Lower Devonian rocks of the Wood Bay Formation from uppermost Devonian–Permian sedimentary successions of the Billefjorden and Gipsdalen groups during early Cenozoic contraction in Garmdalen, i.e., as a local décollement using weak coals and coaly shales of the Billefjorden Group ( Figure 2 and Figure 6a–e). The presence of bedding-parallel décollements between the Wood Bay Formation and the Wordiekammen Formation in Billefjorden is also largely supported from new seismic interpretation in the fjord ( Koehl *et al*., 2023b their Figure 4a–b and f).

A major implication is that the Balliolbreen Fault in Garmdalen most likely did not form as a Carboniferous normal fault because (1) Carboniferous successions thin towards the fault, and (2) the fault does neither downthrow Lower Devonian rocks of the Wood Bay Formation nor uppermost Pennsylvanian–lower Permian rocks of the Wordiekammen Formation ( Figure 8). Based on detailed observation of actual outcrops in northern Garmdalen, the high-angle geometry inferred by previous studies likely arises from collapse of red screes belonging to sedimentary strata of the Hultberget Formation and/or Ebbadalen Formation onto outcrops of lilac-colored Lower Devonian rocks ( Figure 8). The transition between Lower Devonian strata of the Wood Bay Formation and non-collapsed reddish Pennsylvanian screes (Hultberget and/or Ebbadalen formations) in northern Garmdalen actually dips gently to the east (c. 20°; Figure 8) and, therefore, cannot correspond to a high-angle normal fault. This low-angle geometry further supports the initiation of the Balliolbreen Fault in Garmdalen as an Eurekan thrust (e.g., lower western red line in Figure 7) and/or the presence of a low-angle unconformity with onlaps of strata of the Hultberget, Ebbadalen, and Minkinfjellet formations onto the Wood Bay Formation and Billefjorden Group.

### Comparison with the Balliolbreen Fault farther north

**Figure 10.**
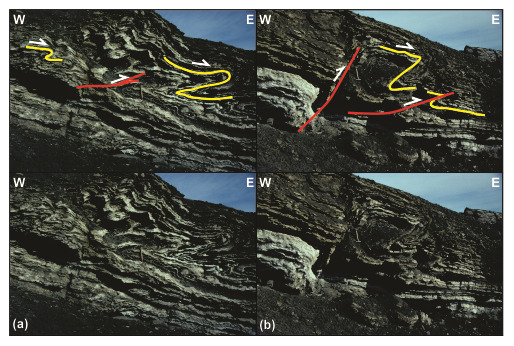
Interpreted (up in each inset) and uninterpreted (down in each inset) outcrop photographs showing top-east folds and thrusts within Pennsylvanian evaporites of the Ebbadalen ( Braathen *et al.*, 2011) or Minkinfjellet Formation ( Dallmann *et al.,* 2004) in Mumien. Photos from Winfried Dallmann.

 In Pyramiden, the Balliolbreen Fault was previously thought to have formed as a Late Devonian reverse fault and to have reactivated as a Carboniferous normal fault ( Bergh *et al*., 2011; Braathen *et al*., 2011; Harland *et al*., 1974; Lamar *et al*., 1982; Lamar *et al*., 1986; Lamar & Douglass, 1995; Smyrak-Sikora *et al*., 2018). However, more recent structural analysis of the fault reveal that the Balliolbreen Fault corresponds to a low-angle, top-west Eurekan thrust comprised of bedding-parallel duplex structures in coal and coaly shale of the Billefjorden Group, and a major anticline with partly overturned eastern limb in Lower Devonian strata ( Koehl, 2021 his Figures 3 and 4a and his supplement S3). Drawing on the similarities between the Balliolbreen Fault in Pyramiden and Garmdalen, it is therefore likely that the pinching-out character of the Hultberget, Ebbadalen, and Minkinfjellet formations in Pyramiden is also partly (completely?) related to early Cenozoic tectonic thinning (smearing), i.e., squeezed out towards the east below the overlying competent carbonate beds of the Wordiekammen Formation. This is further supported by the geometry of sedimentary beds of the Billefjorden Group, and Hultberget and Ebbadalen formations along the Balliolbreen Fault in Pyramiden based on boreholes and several reverse offsets along related minor faults (see Figure 8.2.5 in Aakvik, 1981), by east-verging folds and related minor, top-east thrusts with meter-scale reverse offsets within evaporites of the Ebbadalen/Minkinfjellet Formation in Mumien (see Figure 1 for location; Braathen *et al*., 2011 their Figure 7d; Winfried Dallmann, pers. comm. 2021 – see also Figure 10a–b), and by several other occurrences of tectonic thickening and thinning/smearing of Pennsylvanian evaporite units in Billefjorden ( Harland *et al*., 1988; Ringset & Andresen, 1988).

**Figure 11.**
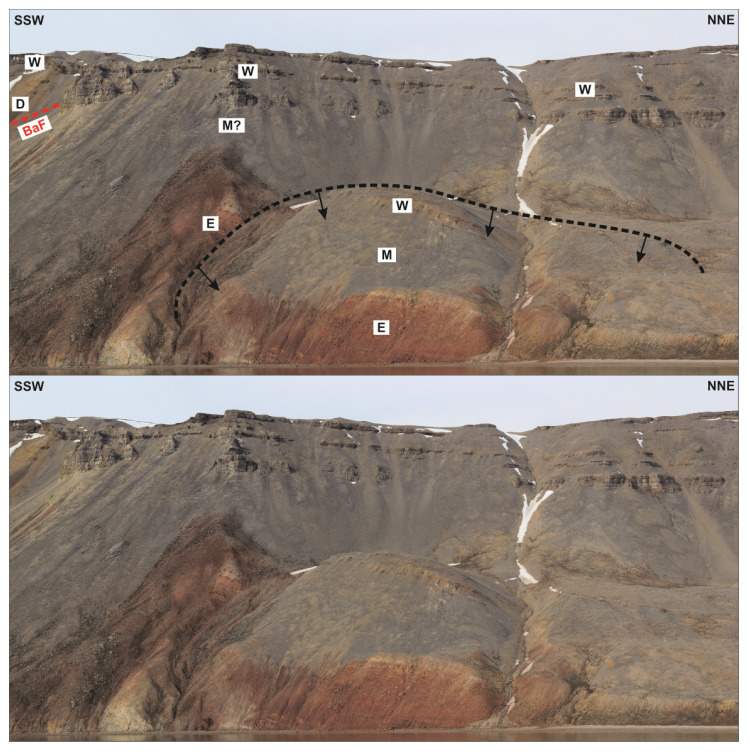
Interpreted (up) and uninterpreted (down) outcrop transect showing a (Quaternary?) landslide block (dashed black line) in the hanging wall of the Balliolbreen Fault (BaF; red line) south of Garmdalen (see Dallmann *et al*., 2004 for exact location) involving Pennsylvanian–lower Permian strata of the Ebbadalen (E), Minkinfjellet (M), and Wordiekammen (W) formations. The black arrows reflect the down-east transport direction of the landslide.

 A major implication is that the minor (c. 10 meters large) apparent normal offset of carbonate beds of the Wordiekammen Formation in northeasternmost Yggdrasilkampen (i.e., southeasternmost Mimerdalen; Figure 4; see Figure 1 for location), which suggests minor, post-earliest Permian normal movements along the fault, does not reflect the overall geometry of the Balliolbreen Fault ( Figure 4). Instead, we re-interpret this minor offset as a late (Quaternary?), gravity-related collapse offset (yellow line in Figure 7). This re-interpretation is supported by the presence of several Quaternary landslides involving carbonate beds of the Wordiekammen Formation in the study area, e.g., southeast of Garmdalen (in the hanging wall of the Balliolbreen Fault; Figure 11; Kuhn *et al*., 2022; Roman *et al.*, 2015), in Alvrekdalen, in Odindalen, in Munindalen, and in southern Wordiekammen ( Dallmann *et al*., 2004; Maher & Braathen, 2011; Michaelsen, 1998). Our re-interpretation is further supported by ^14^C dating below a sag lake in Garmaksla, which suggests a postglacial Quaternary age for the normal offset of the Wordiekammen Formation in Yggdrasilkampen ( Kuhn *et al*., 2022; Roman *et al.*, 2015). This minor Quaternary fault is therefore not representing the Balliolbreen Fault *sensu stricto*, and its geometry cannot be assumed to reflect the actual geometry of the Balliolbreen Fault ( Figure 7). This locality ( Figure 4) was the only one suggesting potential post-earliest Permian normal movement along the Balliolbreen Fault. This re-interpretation therefore further helps disentangling and simplifying the tectonic history of the Balliolbreen Fault by invalidating the possibility of Permian normal faulting along the fault.

**Figure 12.**
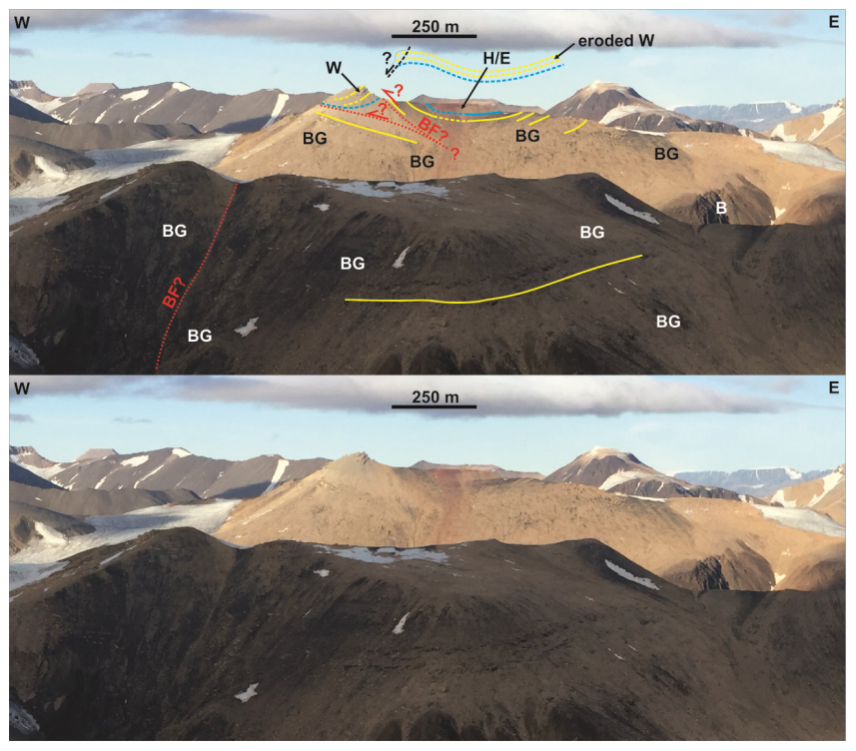
Interpreted (up) and uninterpreted (down) outcrop transect showing bedding surfaces (yellow lines), stratigraphic boundaries (blue lines), and the Balliolbreen Fault (red lines) in Svenbreenhøgda (foreground) and Birger Johnsonfjellet (background). In Birger Johnsonfjellet, red-colored strata of the Ebbadalen (or Hultberget?) Formation (H/E) and Billefjorden Group (BG) are mildly folded into an open syncline and thrust top-west against gently west-dipping carbonates of the Wordiekammen Formation (W). The exact geometry and location of the Balliolbreen Fault is difficult to infer due to the very low extent and quality of the outcrops, but must be low angle and may include moderately dipping splays. Notice the possible relationship of west-dipping outcrops of the Wordiekammen Formation with a potential down-west Quaternary collapse fault (dashed black line). In Svenbreenhøgda, strata of the Billefjorden Group are sub-horizontal and the inferred location of the Balliolbreen Fault (BF) from Dallmann *et al.* (2004). Notice the lack of any good-quality outcrops and of any hard evidence (no obvious offset or strongly deformed rocks) in Svenbreenhøgda, which suggests that the mapping of the fault by previous authors is tentative at best, if the fault is present at all. Proterozoic basement rocks (B) crop out in eastern Birger Johnsonfjellet.

In Mumien, the Balliolbreen Fault juxtaposes Lower Pennsylvanian strata of the Ebbadalen Formation in the hanging wall against uppermost Pennsylvanian–lower Permian rocks of the Wordiekammen Formation in the footwall ( Dallmann *et al*., 2004), therefore suggesting a minimum of 150–200 meters of early Cenozoic, top-west reverse offset in this area. This suggests a similar tectonic evolution to the low-angle brittle faults in Garmdalen ( Figure 6a–e and Figure 8; See also Manby *et al*., 1994). Nevertheless, the Balliolbreen Fault appears to dip steeply eastwards in Mumien (c. 75°; Muñoz-Barrera, 2017 unpublished data) and strata of the Ebbadalen Formation dip gently to the southwest ( Dallmann *et al*., 2004). This contrasts with (1) the low angle (c. 20°) geometry of the Balliolbreen Fault in Garmdalen (if present at all; Figure 8), (2) Carboniferous–Permian strata in Birger Johnsonfjellet (see Figure 1 for location), which appear thrust to the west along a low-angle fault with comparable amounts of offset as in Mumien (Ebbadalen Formation against Wordiekammen Formation; ≥ 150–200 meters) and are folded into a synclinal structure ( Figure 12), and (3) the geometry of strata of the Billefjorden Group in Elsabreen (between Mumien and Pyramiden), which are folded into a west-verging, thrust-related ramp anticline indicating top-west movement ( Stensiö, 1918 his Figure 8). These observations support the formation of the Balliolbreen Fault as a low-angle, early Cenozoic top-west thrust, like in Garmdalen ( Figure 7). Note that the extremely poor quality of outcrops north of Pyramiden does not allow confident mapping of the geometry (apart from its low angle character) and exact location of the Balliolbreen Fault ( Figure 12). A possible explanation for the local high-angle geometry of the Balliolbreen Fault in Mumien and Birger Johnsonfjellet might be a potential splaying attitude of the fault ( Figure 12), or much younger (e.g., Quaternary) collapse/landslide-related faults flattening into the main low-angle thrust ( Kuhn *et al*., 2022 and Figure 12), or a Quaternary gravity-related collapse of strata of the Wordiekammen Formation to the west (see dashed black line in Figure 12). Another possible (complementary) implication of the significant along-strike changes in geometry, kinematics and timing of initiation is that the Balliolbreen Fault is segmented a perpendicular set of faults, which was recently suggested by Koehl (2021) and is supported by recent structural fieldwork ( Koehl *et al*., 2023a) and interpretation of nearshore seismic reflection data in Billefjorden ( Koehl *et al*., 2023b).

### Comparison with the Billefjorden Fault Zone farther south

**Figure 13.**
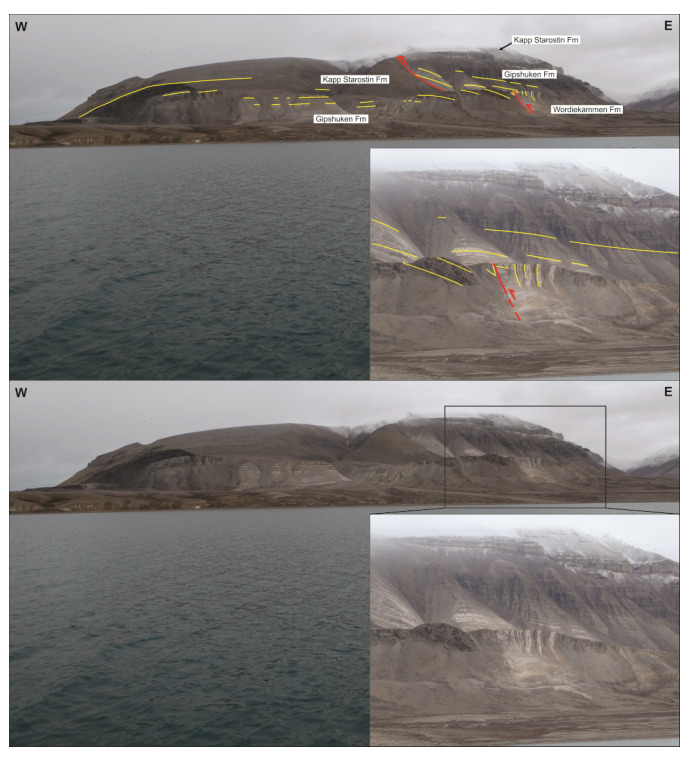
Interpreted (up) and uninterpreted (down) view of the Gipshuken Fault segment of the Billefjorden Fault Zone in Cowantoppen (red lines). Notice how light-colored evaporites of the Gipshuken Formation are thrust top-west along the fault. Photo from Karsten Eig.

**Figure 14.**
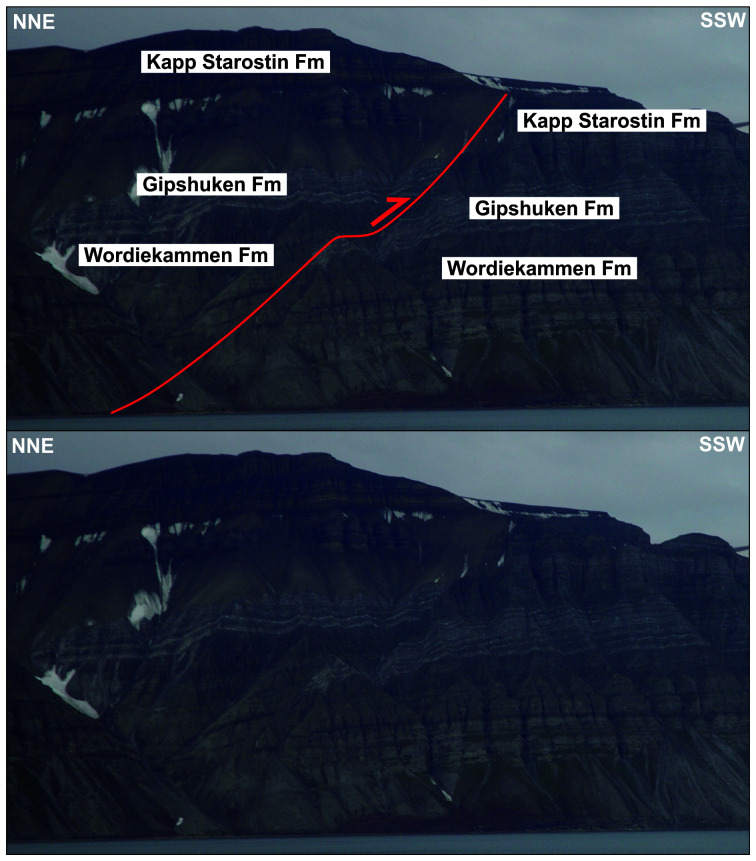
Interpreted (up) and uninterpreted (down) view of the Gipshuken Fault segment of the Billefjorden Fault Zone in Gipshuken (red lines). Uppermost Pennsylvanian–mid Permian strata of the Wordiekammen, Gipshuken, and Kapp Starostin are folded and thrust top-west along the fault. Bedding surfaces are shown as yellow lines. Modified after Koehl (2021). Photos from Eirik Johannessen.

In southeastern Billefjorden, the Billefjorden Fault Zone continues as the Gipshuken Fault, which truncates all strata included lower–middle Permian rocks and displays top-west reverse offsets of up to c. 200 meters in Cowantoppen ( Figure 13) and 350 meters in Gipshuken ( Figure 14; see Figure 1 for locations; Harland *et al*., 1974; Ringset & Andresen, 1988). The overall low-angle geometry and top-west reverse kinematics of the Gipshuken Fault and the truncation of Carboniferous–Permian rocks ( Harland *et al*., 1974; Ringset & Andresen, 1988) indicate that this fault formed as an Eurekan thrust ( Figure 13 and Figure 14). It is therefore likely to represent the southern continuation of the low-angle, east-dipping, top-west Eurekan thrust in Pyramiden ( Koehl, 2021) and Garmdalen (present study; Figure 2, Figure 8, and Figure 7).

In Nordenskiöld Land (location displayed in Figure 1), previous field studies ( Major & Nagy, 1972; Haremo, 1989; Haremo, 1992; Haremo *et al*., 1990) and seismic mapping ( Koehl, 2021) have shown that a gently–moderately east-dipping fault exists at depth and shows comparable top-west reverse offset of Carboniferous–Permian rocks as the Gipshuken Fault in Gipshuken (c. 350 meters; Harland *et al*., 1974). Furthermore, seismic interpretation illustrates the low-angle geometry of the fault ( Bælum & Braathen, 2012 their Figure 8a–b) and suggests that tectonic thickening and thinning/smearing of Devonian–Permian stratigraphic units in the area is related to top-west Eurekan thrusting along the east-dipping fault ( Koehl, 2021). Hence, the east-dipping fault likely formed as a thrust in the early Cenozoic and, therefore, probably represents the southern continuation of the top-west Balliolbreen and Gipshuken faults.

### Implications for the Svalbardian Orogeny

The transitions between the Wood Bay Formation, Billefjorden Group, and Wordiekammen Formation in inner Garmdalen are tectonized into a local bedding-parallel, top-west thrust–décollement system ( Figure 7 and Manby *et al*., 1994 their Figure 12). The significant differences in deformation mode within these three stratigraphic units (strain partitioning), i.e., plastic (folding) for the first one, and brittle (brecciation) for the other two, is believed to reflect rheological contrasts between the units involved. Devonian shales have low Bulk and Shear moduli and therefore are more prone to deform into folds, whereas hard sandstone and carbonate beds of the Billefjorden Group and Wordiekammen Formation are more prone to fracturing because of their high Bulk and Shear moduli (e.g., Molina *et al*., 2017; Zhu *et al*., 2015; Zhang *et al*., 2018). Strain partitioning is also supported by the localization of brittle–ductile, top-west thrusting within coal-rich strata of the Billefjorden Group in eastern Garmdalen ( Figure 6a–e) and in Pyramiden ( Koehl, 2021). Late Devonian Svalbardian contraction is therefore not required to explain deformation differences (plastic versus brittle) between the folded, shale-rich rocks of the Lower Devonian Wood Bay Formation and brecciated sandstones and carbonates of the Billefjorden Group and Wordiekammen Formation in Garmdalen and in Pyramiden.

Some previous studies have suggested that the cleavage patterns in Lower Devonian rocks in Svalbard differ from cleavage in post-Devonian rocks formed to the early Cenozoic Eurekan event. However, cleavage is not a discriminating factor to differentiate tectonic events. For example, transecting cleavage (i.e., cleavage intersecting bedding at an angle to the fold axis) has been shown to form together with oblique fold structures ( Johnson & Woodcock, 1991). In addition, two sets of cleavages with discrete geometries ( Morley, 1986) and/or crosscutting and/or overprinting relationships may also form during a single tectonic event (e.g., Fossen *et al*., 2019; Ghosh *et al*., 1995; Oriolo *et al*., 2022; Treagus & Treagus, 1992). This is also the case for other deformation structures such as foliation, folds, and faults/shear zones, which may also develop various trends simultaneously (e.g., Cobbold, 1976; Ghosh *et al*., 1995; Holdsworth, 1990; Lister & Snoke, 1984). Thus, cleavages in Lower Devonian rocks in Svalbard may as well be early Cenozoic. Further work is needed to test the hypothesis of Late Devonian cleavage in Lower Devonian rocks in Svalbard, e.g., geochronological analysis of mineral precipitation along cleavage.

### Implications for structural inheritance

Nevertheless, although improbable for the above-mentioned reasons, it is possible that the Balliolbreen Fault north of Pyramiden initiated as a Carboniferous normal fault. This is potentially supported by the N–S strike, eastward dip, and alignment along a N–S-trending axis of the inverted Carboniferous normal faults in Odellfjellet–Mumien and top-west Eurekan thrusts in Pyramiden–Garmdalen, Cowantoppen–Gipshuken, and Nordenskiöld Land. However, the length of the system of top-west Eurekan thrusts, which extends from Odellfjellet in the north to Nordenskiöld Land in the south (i.e., at least twice as long as the system of inverted Carboniferous normal faults in Mumien–Odellfjellet; see Figure 1 for location), and its extent well beyond the area potentially affected by east-dipping, inverted Carboniferous normal faults (i.e., from Pyramiden to Nordenskiöld Land), suggest that this is unlikely. Thus, it is more probable that the formation of the system of top-west Eurekan thrusts was controlled by more pervasive, possibly regional structures. Probable candidates are N–S-trending, basement-seated, Caledonian folds and thrusts such as the Atomfjella Antiform in adjacent area of Billefjorden–Ny-Friesland (see location in Figure 1; Gee *et al*., 1992; Harland *et al*., 1992; Witt-Nilsson *et al*., 1998).

### Implications for plate tectonics reconstructions

The present study illustrates clear inconsistencies in the interpretation of the timing of faulting along the Balliolbreen Fault segment of the Billefjorden Fault Zone, which most likely formed in the early Cenozoic rather than in the Late Devonian (e.g., Harland *et al*., 1974) or Carboniferous (e.g., Braathen *et al*., 2011). Our findings cast doubts on the extent and long-lived character of the Billefjorden Fault Zone in Spitsbergen, which was previously thought to represent a major terrane boundary. It is now clear that east-dipping segments of the Balliolbreen Fault in Pyramiden and farther southwards did not form prior to the early Cenozoic (e.g., Figure 7, Figure 8, and Figure 11). This is also probably the case for the Balliolbreen Fault north of Pyramiden (e.g., Figure 12). In addition, recent work in northern Svalbard shows that the Billefjorden Fault Zone dies out in Wijdefjorden (or farther south in Austfjorden; see Figure 1 for locations) and therefore does not represent a major terrane boundary ( Koehl & Allaart, 2021). This suggests that the Balliolbreen Fault did not exist or was restricted to a much narrower area (north of Birger Johnsonfjellet to Odellfjellet or Austfjorden, i.e., ≤ 50 kilometers long instead of several hundreds of kilometers) prior to the early Cenozoic. A major implication is that the model commonly used to explain the accretion of Svalbard’s three basement terranes along N–S-striking faults (e.g., Billefjorden Fault Zone) in the early–mid Paleozoic needs critical revisions. This is partly addressed by recent works, which revealed the presence of continuous, thousands of kilometers long, late Neoproterozoic (Timanian), top-SSW thrust systems that segment N–S-trending structural grain throughout the Barents Sea and Svalbard up to the Fram Strait ( Koehl *et al*., 2022a; Koehl *et al*., 2023b; Koehl, 2024).

### Implications for the mapping of major fault zones during geological fieldwork

 The present study demonstrates that caution must be exerted when mapping major fault zones solely based on fieldwork data. Geological fieldwork only offers an incomplete picture of the geology of an area, especially in deeply eroded Arctic areas. For example, the density of relevant outcrops (in the present case, sparse) and the quality of the examined outcrops both play a significant role in the quality and uncertainty attached to resulting interpretations. Notably, the uncertainty associated with geological field mapping (e.g., sparsity of outcrops, outcrop quality, distance from which the observations were made, and tools used for geological mapping) is too rarely discussed in peer-reviewed works. Such bias may lead to the erroneous inference of major geological events and faults, e.g., Wegener Fault between Greenland and Ellesmere Island (e.g., Oakey & Damaske, 2006), Knoydartian Orogeny in northern UK ( Cutts *et al*., 2009), Antler Orogeny in western USA (e.g., Ketner, 2012), Svalbardian Orogeny ( Koehl *et al*., 2022b), and De Geer Zone in western Svalbard ( Koehl, 2024). Thus, despite providing valuable insights, geological field mapping should only be treated as a possible interpretation of a largely incomplete dataset and, where possible, should be accompanied by or compared with the interpretation of other datasets, such as geophysical and laboratory data, at various scales (from regional to microscopic).

This is no easy task since current educational systems and job market typically promote high degrees of specialization over more interdisciplinary backgrounds. This is not a recent issue ( Einstein, 1954) and further isolates qualified researchers from one another, preventing one mind to comprehend global implications or leading to the development of incompatible/irreconcilable methods and practices across sub-disciplines (e.g., detrital zircon studies – Ibañez-Mejia *et al*., 2018 – and depth derived from pressure estimates – Moulas *et al*., 2013 – both incompatible with geophysical datasets; e.g., Koehl *et al*., 2022a), thus inhibiting scientific progress. Specialized workers must be allowed and encouraged to broaden their horizon and general understanding of the natural sciences. Else, they can neither fully appreciate the flaws and biases, nor the benefit of a specific sub-discipline to the scientific community.

## Conclusions

1) Lower Devonian rocks of the Wood Bay Formation, uppermost Devonian–Mississippian coal-rich sedimentary rocks of the Billefjorden Group, Pennsylvanian–lower Permian strata of the Gipsdalen Group in Garmdalen are all involved in early Cenozoic top-west thrusting and/or folding.

2) The Balliolbreen Fault in Garmdalen is a low-angle fault that formed as a top-west, in place bedding-parallel, early Cenozoic Eurekan thrust, which acted as a partial–local décollements between Lower Devonian rocks of the Wood Bay Formation and uppermost Pennsylvanian–lower Permian strata of the Wordiekammen Formation.

3) During early Cenozoic Eurekan contraction, strain partitioning processes resulted in intense plastic deformation of weak sedimentary units, such as tight folding of Lower Devonian shales and shearing of coal-rich strata of the Billefjorden Group, whereas strong sedimentary beds, such as sandstones of the Billefjorden Group and carbonates of the Wordiekammen Formation were brecciated and mildly folded. Late Devonian Svalbardian contraction is therefore no longer required to explain deformation patterns in this area.

4) The system of east-dipping, early Cenozoic Eurekan thrusts in Billefjorden and Nordenskiöld Land may have formed along widespread zones of weakness in the crust such as Caledonian folds and thrusts.

5) Prior to the early Cenozoic, the Balliolbreen Fault did not exist or had a limited extent ≤ 50 kilometers long, which further invalidates the model commonly used to explain Svalbard’s terrane accretion along thousands of kilometers long, N–S-striking faults like the Billefjorden Fault Zone in the early–mid Paleozoic.

6) Interdisciplinary scientific approaches and educational backgrounds must be further encouraged to avoid critical mistakes in highly specialized fields, such as the geological mapping of major fault zones.

## Data Availability

DataverseNO- Structural field measurements in Proterozoic basement, Devonian, Carboniferous, and Permian rocks in Billefjorden, July 2021. https://doi.org/10.18710/TIIIKX ( Koehl & Stokmo, 2021a). https://doi.org/10.18710/BIJYVO ( Koehl & Stokmo, 2021b). This project contains following data: All the field photographs taken during the July 2021 field expedition to Billefjorden Aerial photographs of the study area are accessible at toposvalbard.npolar.no. Data are available under the terms of the Creative Commons Zero "No rights reserved" data waiver (CC0 1.0 Public domain dedication) ( http://creativecommons.org/publicdomain/zero/1.0/).
