# Supplementary material for: On the Billefjorden fault zone in Garmdalen, central Spitsbergen: implications for the mapping of major fault zones during geological fieldwork and for the tectonic history of Svalbard
Source: Open Res Eur. 2024 Oct 2;4:147. Originally published 2024 Jul 12. [Version 2] doi: 10.12688/openreseurope.17826.2 (PMC11541080; doi:10.12688/openreseurope.17826.2)
Supplement: Peer review history

# Reviewer report: Reviewer response for version 2

**DOI:** 10.21956/openreseurope.20102.r45315

**Published:** 2024-11-06

**Authors:** Mehmet Köküm

**Affiliations:** 1 Firat Universitesi, Elâzığ, Elazığ, Turkey

Dear Author(s), 

 I should emphasize that this manuscript is grounded in extensive fieldwork and a comprehensive literature review. This comprehensive manuscript will make a significant contribution to the literature.

 I ; therefore, would like to offer a few suggestions to further contribute to your work:

 1) In this study, the geometry of the Balliolbreen Fault segment of the Billefjorden Fault Zone is discussed in detail; therefore, the title can be updated to include 'Balliolbreen Fault Segment.

 eg., "On the Balliolbreen Fault Segment of the Billefjorden fault zone..."

 2) I suggest adding another column to Figure 3 to indicate the age range of geological formations (Ma).

 3) On page 9, the first paragraph under the Results header should refer to Figure 5a, b instead of Figure 4a, b etc.

  4) The symbology in Figures 6a and 6d is not as clearly labeled as in Figures 6b and 6c.

 5) In the introduction, the authors mention that the main segments of the Billefjorden Fault Zone include the Odellfjellet Fault, the Balliolbreen Fault, and the Gipshuken Fault. However, in Figure 15, the Odellfjellet Fault appears to be represented as a normal fault.

 For the suggestion outlined above, I recommend minor revisions before acceptance.

 Kind regards,

Is the study design appropriate and does the work have academic merit?

Yes

Is the work clearly and accurately presented and does it cite the current literature?

Yes

If applicable, is the statistical analysis and its interpretation appropriate?

Yes

Are all the source data underlying the results available to ensure full reproducibility?

Yes

Are the conclusions drawn adequately supported by the results?

Yes

Are sufficient details of methods and analysis provided to allow replication by others?

Yes

Reviewer Expertise:

Structural Geology and Active Tectonic.

I confirm that I have read this submission and believe that I have an appropriate level of expertise to confirm that it is of an acceptable scientific standard.

# Reviewer report: Reviewer response for version 2

**DOI:** 10.21956/openreseurope.20102.r44817

**Published:** 2024-10-10

**Authors:** Oleksii V Bartashchuk

**Affiliations:** 1 Kharkiv'skii natsional'nii universitet imeni VN Karazina, Kharkiv, Ukraine

Approved the revised article.

Is the study design appropriate and does the work have academic merit?

Yes

Is the work clearly and accurately presented and does it cite the current literature?

Yes

If applicable, is the statistical analysis and its interpretation appropriate?

Not applicable

Are all the source data underlying the results available to ensure full reproducibility?

Partly

Are the conclusions drawn adequately supported by the results?

Yes

Are sufficient details of methods and analysis provided to allow replication by others?

Yes

Reviewer Expertise:

structural genetic and kinematic analysis of fault and fold tectonics of continental platforms

I confirm that I have read this submission and believe that I have an appropriate level of expertise to confirm that it is of an acceptable scientific standard.

# Reviewer report: Reviewer response for version 1

**DOI:** 10.21956/openreseurope.19273.r43154

**Published:** 2024-09-04

**Authors:** David Lino Vasconcelos

**Affiliations:** 1 Federal University of Campina Grande, Campina Grande,, Brazil

The present manuscript (On the Billefjorden fault zone in Garmdalen, central Spitsbergen: implications for the mapping of major fault zones during geological fieldwork and for the tectonic history of Svalbard) is well-written and partially well-illustrated. This manuscript presents an excellent scientific gap (Nevertheless, despite numerous studies of the Billefjorden Fault Zone, several aspects of its tectonic history remain unclear. Notably, the Balliolbreen Fault displays significant along-strike variations in both inferred kinematics, apparent cross-section geometry, amount of offset, and time of formation). However, some questions need to be address before the acceptance of this study.

 The main question is related to the new results presented in the discussion section. Figures 9, 10, 11, 12, 13, and 14, are exhibited for the first time in the discussion section. These figures are related to the results and not to the discussion. Therefore, I recommend that the authors use the result section exclusively to describe the results and the discussion section exclusively to discuss the results and not to show new results.

 Also, some results are related to a distance view analysis. The results discussed in Figure 9 are based on an analysis of outcrop distance. I wonder how reliable this is. This limitation, if I have understood correctly, needs to be made explicit in the text. In my opinion, only Figure 14 is extremely reliable.

 In addition, several features in figures are not present in the figures. For example, the location of successions 1 and 3. Also, I would like to suggest that the authors localize the site of the photos exhibited in the manuscript (mainly Figures 9, 10, 11, 12, 13, and 14).

 Finally, the resolution of the figures is not good enough. Also, some features presented in the figures are ineligible due to the label size.

 For this reason, I recommend minor to moderate revisions to this manuscript, which can be indexed after the reviews are addressed.

Is the study design appropriate and does the work have academic merit?

Partly

Is the work clearly and accurately presented and does it cite the current literature?

Yes

If applicable, is the statistical analysis and its interpretation appropriate?

Partly

Are all the source data underlying the results available to ensure full reproducibility?

Yes

Are the conclusions drawn adequately supported by the results?

Partly

Are sufficient details of methods and analysis provided to allow replication by others?

Partly

Reviewer Expertise:

I am a geologist with expertise in the area of ​​structural geology applied to the evolution of sedimentary basins.

I confirm that I have read this submission and believe that I have an appropriate level of expertise to confirm that it is of an acceptable scientific standard, however I have significant reservations, as outlined above.

# Response

**Published:** 2024-09-18

**Authors:** Jean-Baptiste Koehl

**Affiliations:** Geosciences, Universitetet i Oslo, Oslo, Oslo, Norway

Dear Prof. Lino Vasconcelos,

 Thank you very much for your input on the manuscript, it is highly appreciated. Here is our reply to your comments. We hope the changes we implemented improve the shortcomings of the manuscript highlighted by your comments and suggestions. Please do not hesitate to contact us shall this not be the case for some comments.  

**Comments by the reviewer -**

**Comment 1:** The main question is related to the new results presented in the discussion section. Figures 9, 10, 11, 12, 13, and 14, are exhibited for the first time in the discussion section. These figures are related to the results and not to the discussion. Therefore, I recommend that the authors use the result section exclusively to describe the results and the discussion section exclusively to discuss the results and not to show new results.

**Author Response:** These figures show geological structures and observations already detailed in previous studies and should therefore not be included in the results. For example, the absence of shales and coals of the Billefjorden Group between the Wood Bay and Wordiekammen formations in inner Garmdalen was first documented with an outcrop photograph by Manby et al. (1994). Figure 9 therefore does not present new results but brings some perspective from previous studies to discuss the structures targeted by the present manuscript.

**Changes:** None required.  

**Comment 2:** Also, some results are related to a distance view analysis. The results discussed in Figure 9 are based on an analysis of outcrop distance. I wonder how reliable this is. This limitation, if I have understood correctly, needs to be made explicit in the text. In my opinion, only Figure 14 is extremely reliable.

**Author Response: ** Disagreed. Figure 9–14 do not show new results, but illustrate facts from previous studies (e.g., Manby et al., 1994 for Figure 9; Braathen et al., 2011 for Figure 10; etc.). Thus, these figures are mentioned only in the discussion. However, the reviewer raises a good point about Figure 9, which could benefit the manuscript better if mentioned in the result chapter.

Changes: Included reference to Figure 9 in the result chapter.  

**Comment 3:** In addition, several features in figures are not present in the figures. For example, the location of successions 1 and 3. Also, I would like to suggest that the authors localize the site of the photos exhibited in the manuscript (mainly Figures 9, 10, 11, 12, 13, and 14).

**Author Response: ** Agreed, although the location of successions 1–3 is displayed in Figure 2.

Changes: Changed the color of the location of succession 1–3 and added the location of Figures 8 and 9 in Figure 2a, added a new Figure 2b with the location of Figure 5a, 6a and 6d, and added the location of Figures 10–14 in Figure 1b.  

**Comment 4:** Finally, the resolution of the figures is not good enough. Also, some features presented in the figures are ineligible due to the label size.

**Author Response:** Agreed.

Changes: Asked the journal to enlarge the figure as much as possible and submitted high-resolution versions of the figures to an Open Access data repository (DataverseNO). Added reference to the dataset in the manuscript.

# Reviewer report: Reviewer response for version 1

**DOI:** 10.21956/openreseurope.19273.r42739

**Published:** 2024-08-23

**Authors:** Oleksii V Bartashchuk

**Affiliations:** 1 Kharkiv'skii natsional'nii universitet imeni VN Karazina, Kharkiv, Ukraine

I fully agree with the authors' main conclusion: "Interdisciplinary scientific approaches and educational backgrounds must be further encouraged to avoid critical mistakes in highly specialized fields, such as the geological mapping of major fault zones" as an ideal to strive for. To make the article scientifically sound:

 1- supplement the conclusions with an important thesis about the natural phenomenon of structural-dynamic and kinematic inversion of faults. It is obvious from the presented material that the same fault systems, activated in structural (folded) floors in different epochs of orogenesis in different geodynamic regimes, change their genetic type and, accordingly, diagnostic features during field mapping.

 2- add a compilation of figures from the cited works of seismic studies, which confirm the correctness of the conclusions about the Cenozoic age and thrust nature of the Billefjorden Fault Zone.

 3- clarify the legend (symbols) in Figure 1.

Is the study design appropriate and does the work have academic merit?

Yes

Is the work clearly and accurately presented and does it cite the current literature?

Yes

If applicable, is the statistical analysis and its interpretation appropriate?

Not applicable

Are all the source data underlying the results available to ensure full reproducibility?

Partly

Are the conclusions drawn adequately supported by the results?

Yes

Are sufficient details of methods and analysis provided to allow replication by others?

Yes

Reviewer Expertise:

structural genetic and kinematic analysis of fault and fold tectonics of continental platforms

I confirm that I have read this submission and believe that I have an appropriate level of expertise to confirm that it is of an acceptable scientific standard.

# Response

**Published:** 2024-09-18

**Authors:** Jean-Baptiste Koehl

**Affiliations:** Geosciences, Universitetet i Oslo, Oslo, Oslo, Norway

Dear Prof. Bartashchuk, thank you very much for your input on the manuscript, it is highly appreciated. Here is our reply to your comments. We hope the changes we implemented improve the shortcomings of the manuscript highlighted by your comments and suggestions. Please do not hesitate to contact us shall this not be the case for some comments.  

**Comments by the reviewer**

**Comment 1:** To make the article scientifically sound: 1- supplement the conclusions with an important thesis about the natural phenomenon of structural-dynamic and kinematic inversion of faults. It is obvious from the presented material that the same fault systems, activated in structural (folded) floors in different epochs of orogenesis in different geodynamic regimes, change their genetic type and, accordingly, diagnostic features during field mapping.

Response: Agreed, although the along-strike changes in the present case of the Balliolbreen Fault are likely related to strain partitioning in the various lithostratigraphic units, which have highly varying rheology, e.g., the shale-rich Wood Bay Formation and coal-rich member of the Billefjorden Group are intensely deformed near the fault, e.g., Figure 6a–c and Figure 8 in the present study, and Figures 3 and 4a–b in Koehl (2021), whereas strong carbonate beds of the Wordiekammen Formation are hardly deformed, e.g., Figure 8 in the present study. If this is what is suggested by the reviewer, then these points are already discussed in the manuscript (see section “Implications for the Svalbardian Orogeny” pp 19–21).

Changes: None yet. Awaiting further instructions/clarification by the reviewer if necessary.  

**Comment 2:** 2- add a compilation of figures from the cited works of seismic studies, which confirm the correctness of the conclusions about the Cenozoic age and thrust nature of the Billefjorden Fault Zone.

Response: Agreed.

Changes: Added Figure 5g from Koehl (2021) as new Figure 15.  

**Comment 3:** 3- clarify the legend (symbols) in Figure 1.

Response: Agreed.

Changes: Updated Figure 1b.
